# Cleavable hairpin beacon-enhanced fluorescence detection of nucleic acid isothermal amplification and smartphone-based readout

**DOI:** 10.1038/s41598-020-75795-y

**Published:** 2020-11-02

**Authors:** Xiong Ding, Kun Yin, Ziyue Li, Vikram Pandian, Joan A. Smyth, Zeinab Helal, Changchun Liu

**Affiliations:** 1 Department of Biomedical Engineering, University of Connecticut Health Center, 263 Farmington Avenue, Farmington, CT 06030 USA; 2 Electrical and Computer Engineering, University of Texas at Austin, Austin, TX 78712 USA; 3 Connecticut Veterinary Medical Diagnostic Laboratory, Department of Pathobiology and Veterinary Science, University of Connecticut, Storrs, CT 06269 USA

**Keywords:** Analytical biochemistry, Sensors and probes, Assay systems, Biomedical engineering

## Abstract

Fluorescence detection of nucleic acid isothermal amplification utilizing energy-transfer-tagged oligonucleotide probes provides a highly sensitive and specific method for pathogen detection. However, currently available probes suffer from relatively weak fluorescence signals and are not suitable for simple, affordable smartphone-based detection at the point of care. Here, we present a cleavable hairpin beacon (CHB)-enhanced fluorescence detection for isothermal amplification assay. The CHB probe is a single fluorophore-tagged hairpin oligonucleotide with five continuous ribonucleotides which can be cleaved by the ribonuclease to specifically initiate DNA amplification and generate strong fluorescence signals. By coupling with loop-mediated isothermal amplification (LAMP), the CHB probe could detect *Borrelia burgdorferi* (*B. burgdorferi*)* recA* gene with a sensitivity of 100 copies within 25 min and generated stronger specific fluorescence signals which were easily read and analysed by our programmed smartphone. Also, this CHB-enhanced LAMP (CHB-LAMP) assay was successfully demonstrated to detect *B. burgdorferi* DNA extracted from tick species, showing comparable results to real-time PCR assay. In addition, our CHB probe was compatible with other isothermal amplifications, such as isothermal multiple-self-matching-initiated amplification (IMSA). Therefore, CHB-enhanced fluorescence detection is anticipated to facilitate the development of simple, sensitive smartphone-based point-of-care pathogen diagnostics in resource-limited settings.

## Introduction

Nucleic acid isothermal amplification testing has become a promising alternative to conventional polymerase chain reaction (PCR) in biomedical applications, such as pathogen detection^1,2^, clinical molecular diagnostics^3^, screening genetically modified organisms^4^, and fundamental bioanalytical study^5^ due to its simplicity, rapidity, cost-effectiveness, and high sensitivity. Currently, a variety of isothermal nucleic acid amplification methods have been developed, including the loop‐mediated isothermal amplification (LAMP)^6^, isothermal multiple-self-matching-initiated amplification (IMSA)^7^, recombinase polymerase amplification (RPA)^8^, rolling circle amplification (RCA)^9^, and nucleic acid sequence‐based amplification (NASBA)^10^. Among them, the LAMP assay is the most widely used isothermal amplification technology for point-of-care pathogen diagnostics.

The current detection approaches for LAMP assay include fluorescence, colorimetry, turbidity, gel electrophoresis, electrochemical methods, and lateral flow dipstick^11^. In these methods, the sequence-specific fluorescence detection utilizing energy-transfer-tagged oligonucleotide probes is one of the most sensitive and specific methods^12^. The reported energy-transfer-tagged probes for LAMP assays include DARQ probe^13^, FBD probe^14^, QUASR probe^15^, OSD probe^16^, and molecular beacon^17,18^. However, these probes either potentially interfere isothermal amplification or generate nonspecific signals to lower the fluorescence change^19^. Recently, a ribonuclease-dependent cleavable beacon primer (CBP) has been developed for fluorescence LAMP detection of single nucleotide mutation (SNM) by our lab^20^. But the CBP shows high background at elevated temperature (e.g., 61 °C) due to its linear structure^20^ and is not suitable to real-time monitor the LAMP reaction which usually takes place at 60–65 °C^21^.

Recently, there has been a major push to use smartphone technology for infectious disease detection instead of expensive equipment, enabling smartphone-based point-of-care diagnostic applications^22^. For example, Song et al.^23^ combined the bioluminescent real time reporter (BART) of LAMP with a smartphone to rapidly quantify Zika virus and spatiotemporally map the disease. Damhorst et al.^24^ developed a smartphone-based LAMP platform for HIV virus detection from whole blood samples. Yin et al.^25^ used the smart cup for synergistically enhanced colorimetric LAMP detection of HPV virus. Rodriguez-Manzano et al.^26^ combined the LAMP assay with smartphone-based detection to develop a rapid, portable and affordable lab-on-a-chip platform for the detection of mobilized colistin resistance. However, their detection signals were not specifically generated by the sequence-specific probes, which potentially leads to false positive results. Due to the low fluorescence signal of the fluorescence probes, it still remains a challenge to adopt a smartphone to directly detect the fluorescence signals generated by the energy-transfer-tagged oligonucleotide probes.

In this study, we developed a novel energy-transfer-tagged oligonucleotide probe, termed cleavable hairpin beacon (CHB), to create a highly sensitive and specific nucleic acid isothermal amplification assay with an improved fluorescence change, enabling smartphone-based point of care detection. The CHB is a single fluorophore-tagged hairpin oligonucleotide with five continuous ribonucleotides which can be cleaved by the ribonucleases to simultaneously initiate rapid nucleic acid amplification and generate sequence-specific fluorescence. As an application demonstration, the CHB probe was coupled with LAMP assay, termed CHB-LAMP, to detect the DNA of *Borrelia burgdorferi* (*B. burgdorferi*), the causative agent of Lyme disease. In addition, the CHB probe was used for the IMSA assay to detect a specific gene sequence (VP1 gene) of Enterovirus (EV) 71 virus, the leading causative pathogen of hand, foot, and mouth disease (HFMD).

## Results and discussion

### CHB-LAMP assay

**Figure 1:**
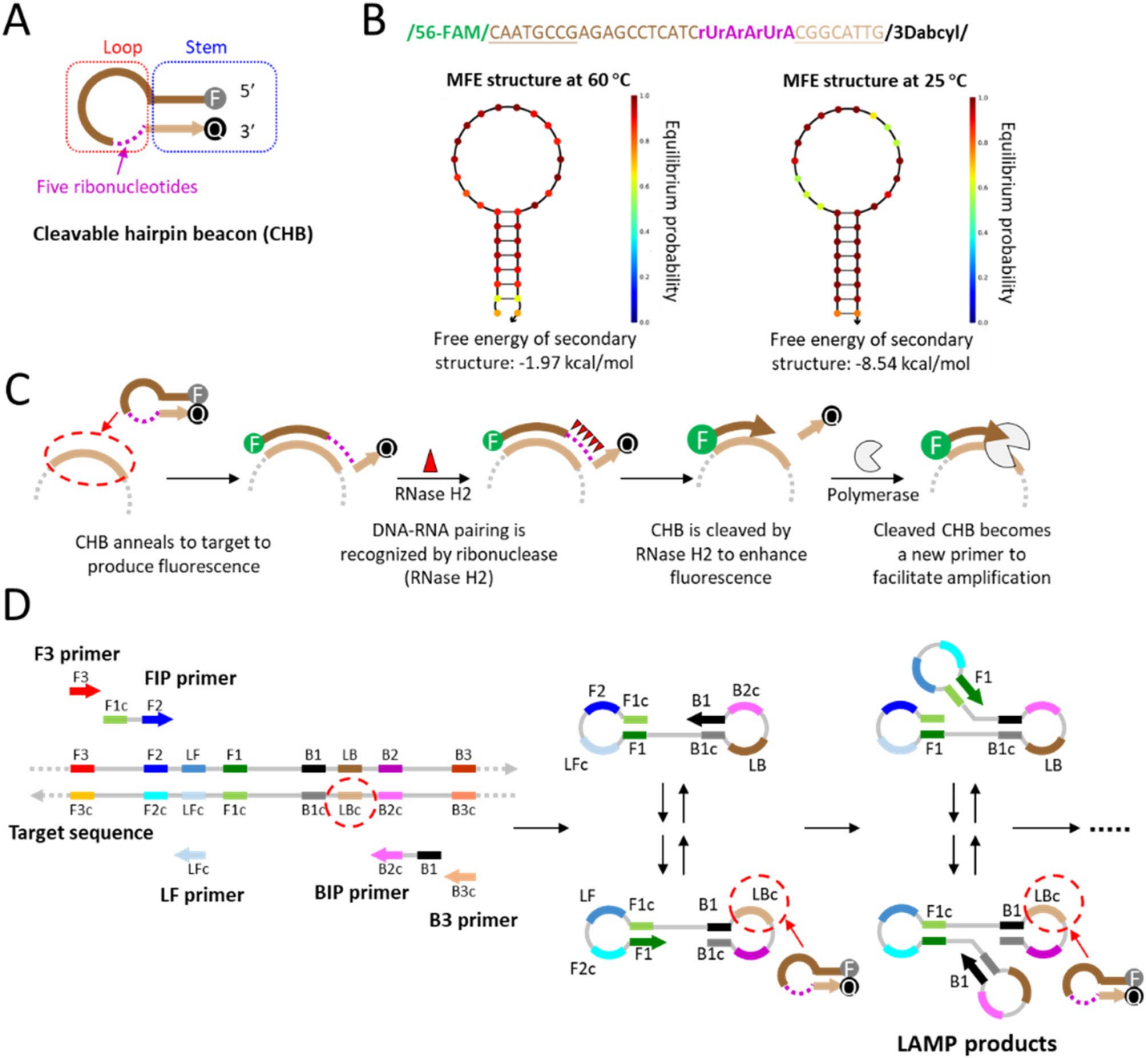
CHB probe design and CHB-LAMP assay. (**A**) Structure design of the CHB probe. (**B**) The CHB probe sequence to amplify *B. burgdorferi recA* gene by the CHB-LAMP and its minimum free energy (MFE) analysis using the software NUPACK (Caltech) with concentrations of 0.8 μM CHB, 70 mM Na^+^ and 6 mM Mg^2+^. (**C**) Schematic of the target sequence recognition and enhanced fluoresce detection of the CHB probe. (**D**) Principle of the CHB-LAMP assay. In the assay, LAMP’s loop backward (LB) primer was used to design the CHB probe.

As shown in Fig. 1A, the CHB probe typically containing loop and stem parts is a single fluorophore-tagged (fluorophore 6-FAM at the 5′-end and quencher DABCYL at the 3′-end) hairpin oligonucleotide with five continuous ribonucleotides close to its stem in the 3′ direction. The loop size of the CHB probe is 16–18 nucleotides (nt). To ensure the stable hairpin structure at elevated temperature (e.g., 60 °C), the stem size of the CHB probe was increased to 8 nt, which is longer than that of conventional molecular beacons (e.g., 6 nt)^27^. The loop sequence of the CHB probe is completely target-specific. To study the formation of the hairpin structure, the minimum free energy (MFE) of the CHB probe was analyzed by using an online NUPACK software. Figure 1B shows the MFE analysis result for the CHB probe designed to detect *B. burgdorferi recA* gene of tick DNA by the CHB-LAMP assay. Due to the increased stem length, the CHB probe can maintain the stable hairpin structure at both elevated temperature (e.g., 60 °C) (MFM = − 1.97 kcal/mol) and room temperature (e.g., 25 °C) (MFM = − -8.54 kcal/mol), which significantly reduces background fluorescence and enables real-time quantitative isothermal amplification detection.

Once the CHB probe anneals to the target DNA sequence, its hairpin structure is destroyed due to the formation of the hybrid DNA-RNA pairing in its ribonucleotide sites (Fig. 1C). The ribonuclease of RNase H2 can specifically recognize the DNA-RNA pairing and hydrolyse the phosphodiester bonds of RNA to create a free 3′-OH end. The introduction of five continuous ribonucleotides improves the cleavage efficiency of the CHB probe by the RNase H2^28^. The cleaved CHB probe can serve as a new primer to further accelerate the amplification process. Figure 1D showed the principle of the CHB-LAMP assay. Since previous study showed that loop primers plays a crucial role in accelerating LAMP amplification reaction^29^, the loop backward (LB) primer was targeted to design the CHB probe in our CHB-LAMP assay. During the CHB-LAMP reaction, strong fluorescence signal was produced due to the specific cleavage of the CHB probe (Fig. 1D).

### Optimization of the CHB-LAMP assay

As proof-of-concept assay, the *B. burgdorferi recA* gene sequence was targeted to develop real-time CHB-LAMP. Firstly, we used conventional real-time fluorescence LAMP with EvaGreen dye to optimize the LAMP reaction conditions, such as reaction temperature, sequences of primers, and concentration of different compositions (e.g., primers, MgSO_4_, dNTPs). As shown in Supplementary Figs. S1 and S2, the optimal reaction temperature was 60 °C and the optimal reaction mixture contained 4 mM MgSO_4_, and 1.6 mM dNTPs. Next, we designed the CHB probe to develop the CHB-LAMP assay. As shown in Supplementary Fig. S3A, the performance of the real-time CHB-LAMP was influenced by the concentration of the ribonuclease RNase H2. In our experiment, the concentration of 2.0 U/mL was optimal because of its strong fluorescence signal. The success of developing real-time CHB-LAMP may be attributed to the extended stem (e.g., up to 8 nt) of the CHB probe that stabilizes the hairpin structure at elevated temperature (e.g., 60 °C), thereby dramatically decreasing the background fluorescence.

To further confirm the specificity of the fluorescence signal generated in the CHB-LAMP assay, a denaturing 15% polyacrylamide gel electrophoresis (PAGE) with 8 M urea was carried out to analyse the amplified products. As shown in Supplementary Fig. S3B, self-probed fluorescence signal was readily observed from the amplicons of the CHB-LAMP with the RNase H2 and template, whereas not in the CHB-LAMP without either RNase H2 or template. These results demonstrated that the fluorescence produced in the CHB-LAMP was highly specific to target DNA. In addition, it further confirmed that the cleaved CHB indeed served as new LB primer to participate in the enzymatic amplification.

### Sensitivity of the CHB-LAMP assay

**Figure 2:**
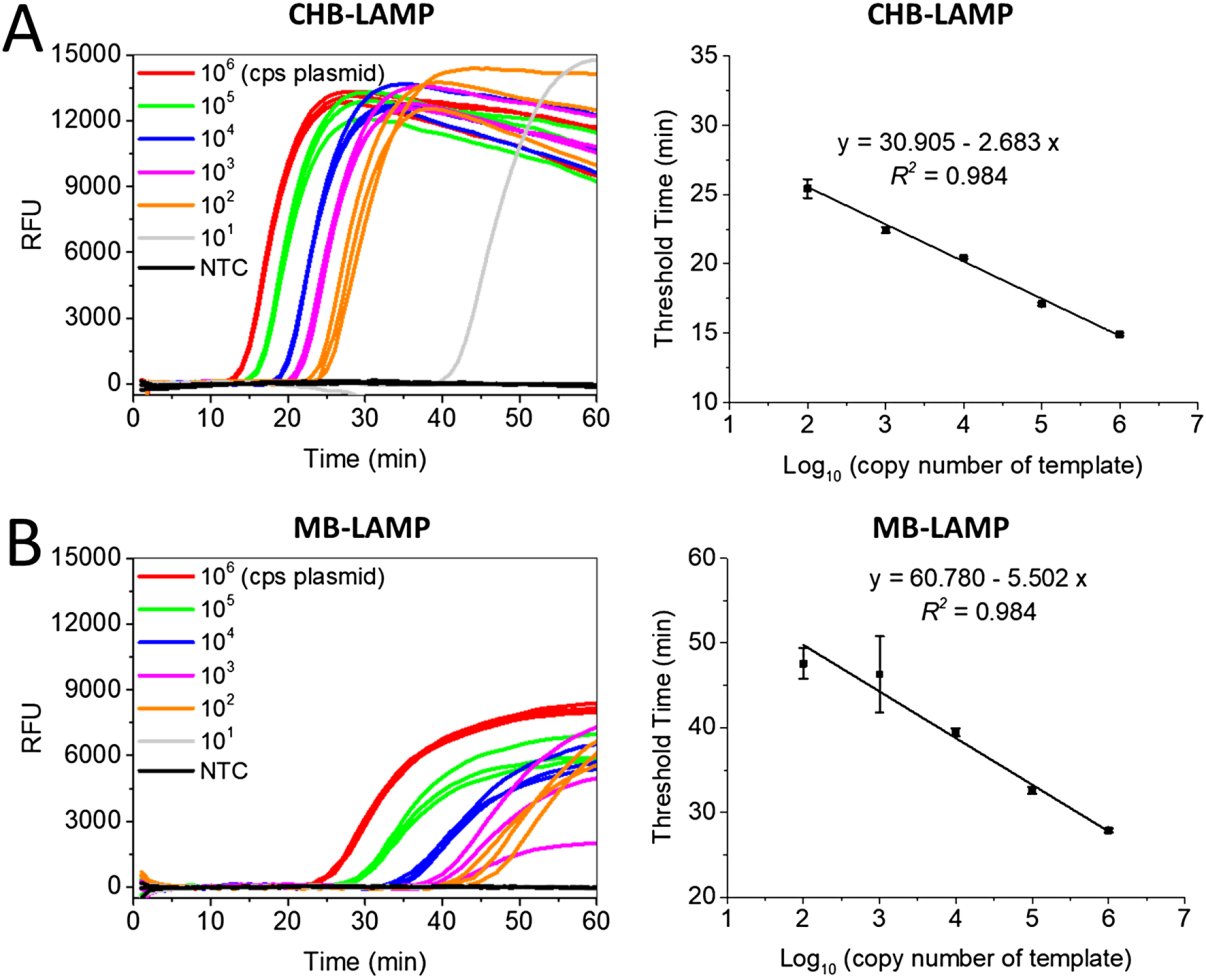
Real-time fluorescence quantitative detection of *B. burgdorferi* DNA by (**A**) CHB-LAMP and (**B**) MB-LAMP assays. Left, the real-time fluorescence curves; right, the linear relationship between the threshold time and the log_10_ of template’s copy number. The plasmids (*recA* gene sequence included) with the copy number (cps) ranging from 10^6^ to 10^1^ were used as the templates. *NTC* non-template control. Each error bar represents the standard deviation for three replicates.

To determine the sensitivity of the CHB-LAMP assay, we tested a tenfold serial dilution of plasmids containing *B. burgdorferi recA* gene by the real-time fluorescence CHB-LAMP assay. For comparison, the MB-LAMP and real-time PCR assays were run in parallel. As depicted in Fig. 2A, the CHB-LAMP was able to detect as few as 100 copies of target DNA with an excellent linearity (*R*^2^ = 0.984), within about 25 min, which was almost two times faster than that of the conventional MB-LAMP assay (Fig. 2B). Figure 2A also indicated that the CHB-LAMP assay was more reproducible, with relative standard deviation (RSD; n = 3) ranging from 0.44 to 2.66%, compared to the MB-LAMP assay with RSD ranging from 0.91 to 9.72% (Fig. 2B). In addition, the CHB-LAMP produced an almost two times greater fluorescence signal than the MB-LAMP assay, enabling us to directly adapt a smartphone for fluorescence signal readout and agent detection at the point of care. Further, the CHB-LAMP assay achieved a comparable sensitivity (e.g., 100 copies of target DNA) with that of the real-time PCR method (Supplementary Figs. S4 and S5). Thus, our CHB-LAMP showed high sensitivity with better quantitative ability compared to the conventional MB-LAMP.

### CHB-enhanced fluorescence detection and smartphone readout

**Figure 3:**
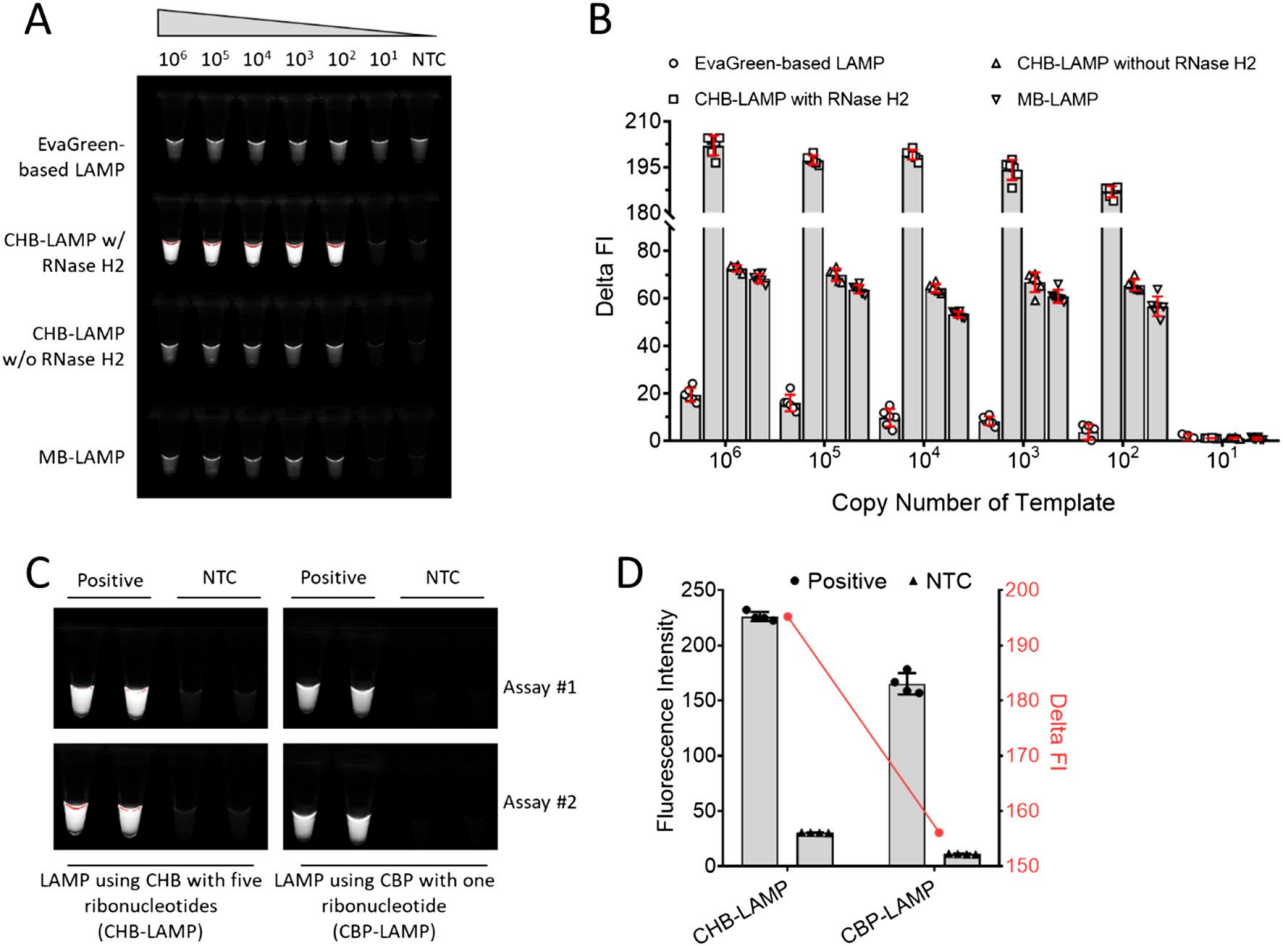
Endpoint fluorescence detection of the EvaGreen-based LAMP, CHB-LAMP with RNase H2, CHB-LAMP without RNase H2, and MB-LAMP after 60-min amplification of various copies of plasmid DNA targets. (**A**, **B**) The fluorescence image and fluorescence intensity (FI) comparison of different methods. Each error bar represents the standard deviation for six replicates. (**C**, **D**) The fluorescence image and FI comparison of the CHB-LAMP and CBP-LAMP assay. Each error bar represents the standard deviation for four replicates. Positive, the CHB-LAMP or CBP-LAMP reaction with 10^5^ copies of plasmids (300-bp recA gene inserted). *NTC* non-template control. The images were captured by using the Bio-Rad Gel Imaging System. The FI was calculated using the Image J software. The delta FI was defined as the FI change between positive and the NTC (Delta FI = FI_Positive_–FI_NTC_).

To achieve sensitive, cost-effective point of care detection in the context of mobile health, it is critical to generate a strong enough fluorescence signal that can be detected with a smartphone. We compared the endpoint fluorescence detection of the CHB-LAMP with other methods: (i) conventional EvaGreen-based LAMP, (ii) CHB-LAMP without RNase H2, and (iii) MB-LAMP. As shown in Fig. 3A, the CHB-LAMP with the RNase H2 significantly enhanced the endpoint fluorescence change between positive and NTC. Particularly on detecting low copy number of targets (e.g., 100 copies targets), the enhanced fluorescence change (delta FI in Fig. 3B) by the CHB-LAMP with RNase H2 was 187.0 ± 1.8 (n = 6), which was more than 48, 1.8, and 2 times higher than those of the EvaGreen-based LAMP, CHB-LAMP without RNase H2, and MB-LAMP, respectively. Further, the amplified products were subjected to denaturing PAGE (Supplementary Fig. S6), confirming the highly specific amplification detection.

We also compared the CHB-LAMP with the previous CBP-LAMP assay^20^ in which the CBP probe only contained one ribonucleotide. As shown in Fig. 3C,D, the fluorescence change [Delta fluorescence intensity (FI) = 195.6] in the CHB-LAMP was about 1.3 times higher than that (Delta FI = 154.1) of the CBP-LAMP assay. This is likely attributed to the introduction of more ribonucleotides in the CHB probe, which increases the cleavage efficiency of the RNase H2 during isothermal amplification.

**Figure 4:**
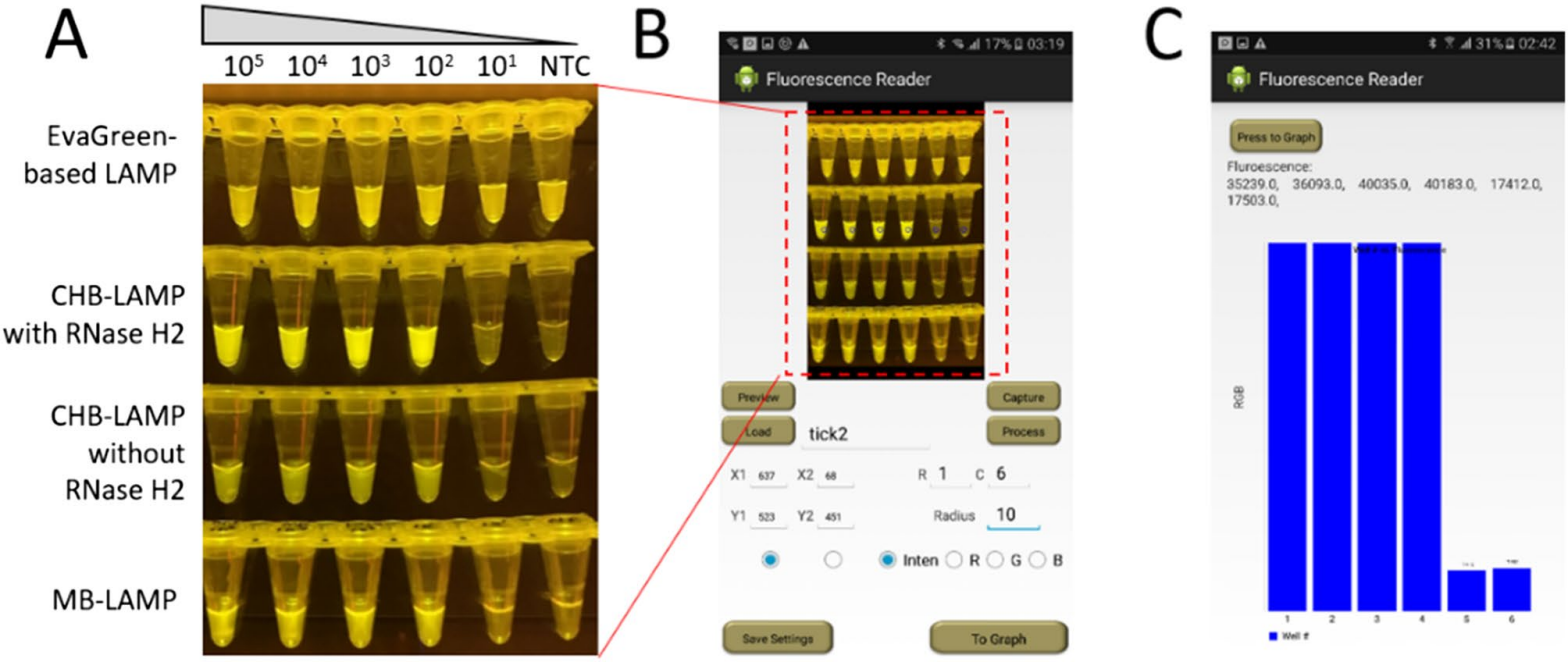
Smartphone-based fluorescence readout of LAMP products of different methods. (**A**) Image of the LAMP products taken by smartphone. (**B**) Screenshot of the app interface for imaging/parameter setting. (**C**) Screenshot of the app interface for fluorescence quantitative readout. Well #1–#5, the reactions with 10^5^ to 10^1^ copies of the plasmid templates. Well #6, non-template control (NTC).

To further adapt the smartphone for fluorescence detection at the point of care, we developed a smartphone app which we entitled “Fluorescence Reader” to quantitatively detect the fluorescence intensity of the CHB-LAMP products. After 60-min amplification at 60 °C, the tubes were firstly placed on a portable LED blue light illuminator. Next, the programmed smartphone was used to record the fluorescence image, analyse the fluorescence signals and quantitatively report the fluorescence intensity (Fig. 4). As shown in Fig. 4A, the CHB-LAMP products with RNase H2 showed the most remarkable fluorescence change between positive and negative compared with the EvaGreen-based LAMP, CHB-LAMP without RNase H2, and MB-LAMP. To quantify the fluorescence, the average green value of each tube in the photo was calculated automatically by the smartphone app (Fig. 4B,C). As shown in Fig. 4C, the green value of each CHB-LAMP reaction tube could be quantitatively reported by the app without need for complex optical equipment. Therefore, the enhanced fluorescence detection of the CHB-LAMP assay was beneficial for smartphone readout, enabling point-of-care, cost-efficient pathogen diagnostics in resource-limited settings.

### Performance of the CHB-LAMP assay on real sample test

**Figure 5:**
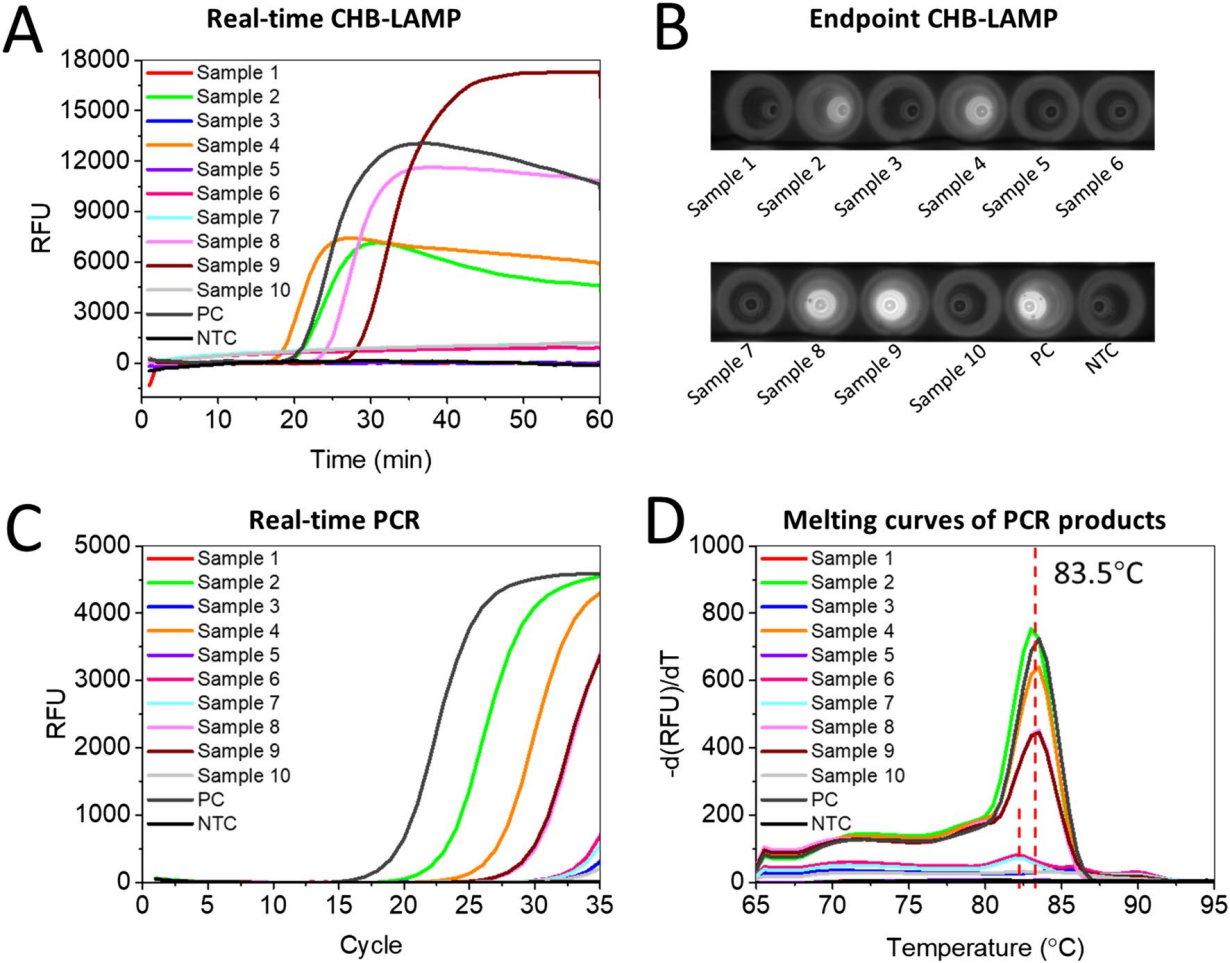
Detection of *B. burgdorferi* DNA extracted from ten tick samples by both the CHB-LAMP and PCR methods. (**A**) Real-time fluorescence CHB-LAMP assay. (**B**) Endpoint CHB-LAMP detection in a 96-well plate. Images were taken on the Bio-Rad Gel Imaging System. (**C**) EvaGreen-based real-time PCR assay. (**D**) Melting curves of the PCR products from (**C**). *PC* positive control with 10^6^ copies plasmid templates, *NTC* non-template control.

Lyme disease is one of the most common tick-borne illnesses that is caused by *B. burgdorferi *sensu lato transmitted by infected Ixodes scapularis ticks, commonly known as deer ticks. Rapid and accurate detection of *B. burgdorferi* in tick species plays an important role in assessing the epidemiology and risk of tick-borne diseases, and predicting the disease outcome for tick-bitten patients^30,31^. Conventional methods (e.g., PCR, culture) for *B. burgdorferi* detection are labour-intensive and time-consuming, which is not suitable for rapid, sensitive, field-deployable detection. To further verify the application potential in testing real samples, we have adapted the CHB-LAMP to detect *B. burgdorferi* gene in the DNA samples extracted from ten different tick species. As shown in Fig. 5A,B, four positive samples and six negative samples were accurately detected by both real-time CHB-LAMP and endpoint CHB-LAMP assays, which were well in agreement with the real-time PCR results (Fig. 5C,D).

### Versatility of the CHB probe in the IMSA assay

**Figure 6:**
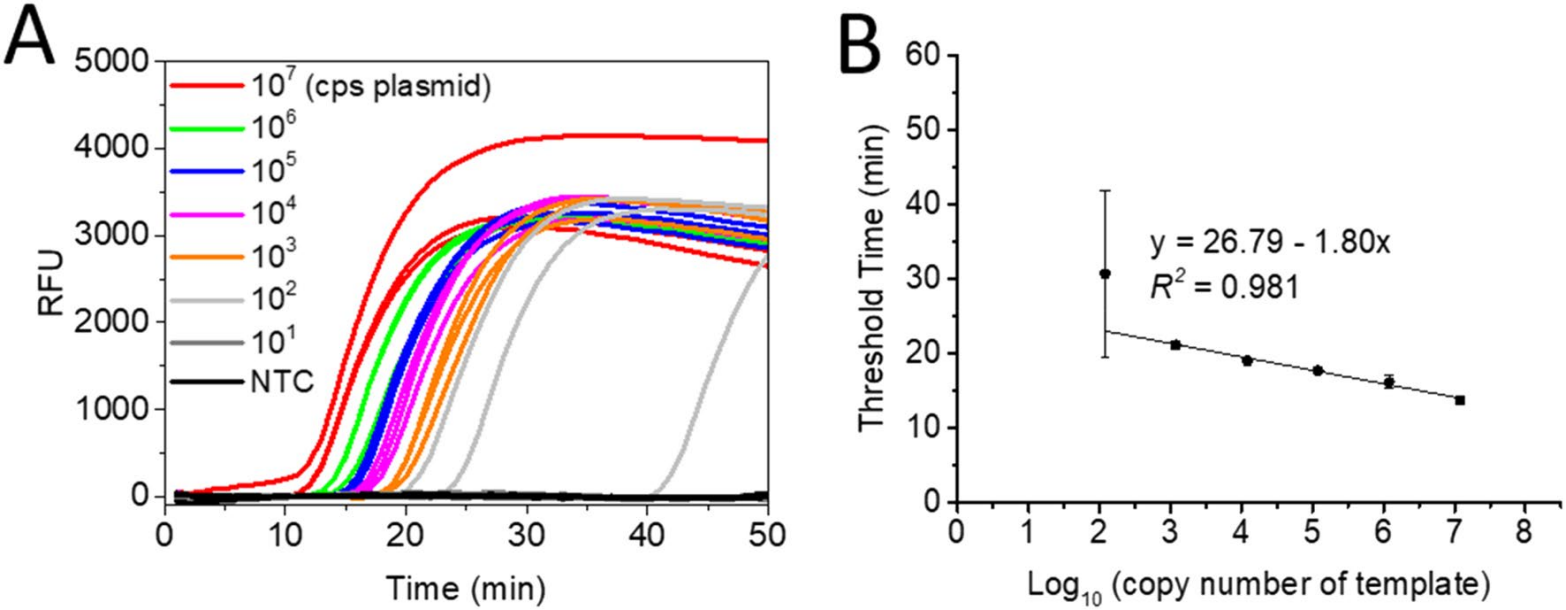
CHB-IMSA assay of the tenfold serial dilution of plasmid bearing EV71 VP1 gene. (**A**) Real-time fluorescence curves of the CHB-IMSA assay to detect the plasmids (300-bp VP1 gene included) with the copy number (cps) ranging from 10^7^ to 10^1^ copies per reaction. (**B**) The linear relationship between the threshold time and the log_10_ of DNA copy number. *NTC* non-template control. Each error bar represents the standard deviation for three replicates.

To evaluate the versatility of the CHB probe in other isothermal amplifications, we coupled the CHB probe with the IMSA assay to detect a specific gene sequence (VP1 gene) of Enterovirus (EV) 71 virus, which is the leading causative pathogen of hand, foot, and mouth disease (HFMD)^32–34^. The principle of the CHB-IMSA assay is given in Supplementary Fig. S7. Since the stem primers of the IMSA assay play a critical role in initiating a rapid and highly efficient amplification detection^7^, we designed the CHB probe to target the stem primer of SteR. To optimize the structure design of the CHB probe, its minimum free energy was analysed at different temperature. The simulation results of the CHB probe showed that it maintained a hairpin structure at both 63 °C (MFM = − 2.04 kcal/mol) and 25 °C (MFM = − 9.17 kcal/mol) (Supplementary Fig. S7A). To further optimize the CHB-IMSA assay, we tested different concentrations of the CHB probe ranging from 0.4 to 1.6 μM and obtained an optimal concentration of 0.4 μM (Supplementary Fig. S8). To determine the sensitivity, we detected tenfold serial dilution of plasmids bearing VP1 gene sequence by the CHB-IMSA assay. Our results showed that the CHB-IMSA can detect as low as 100 copies of the EV71 VP1 gene (Fig. 6) with a good linearity (*R*^2^ = 0.981) between the threshold time and the log_10_ of template’s copy number (from 10^7^ to 10^3^ copies). These results demonstrate that the CHB probe is compatible with the IMSA assay and has great potential to be utilized in different nucleic acid amplification detection.

## Conclusions

In this study, we developed a novel, cleavable, energy-transfer-tagged probe for rapid, sensitive and specific nucleic acid detection with enhanced fluorescence signals by coupling with isothermal amplifications. The CHB-LAMP was successfully demonstrated to detect *B. burgdorferi* DNA in samples extracted from ticks. Compared with conventional MB^17^ and the previously reported CBP probe^20^, our CHB probe provides several advantages for nucleic acid amplification detection: (i) Enhanced fluorescence signal readout. The introduction of multiple ribonucleotides in the CHB probe increases the cleavage efficiency and produces higher fluorescence signal, and furthermore, the longer stem of the CHB probe ensures it to maintain a stable hairpin structure at elevated temperature (e.g., 60 °C), significantly reducing background fluorescence and enabling highly sensitive, real-time fluorescence quantitative detection. By combing microfluidics- or droplet-based detection technology, the CHB-LAMP can be adapted for absolute quantification of nucleic acids. (ii) Shorter detection time. The CHB probe facilitated nucleic acid isothermal amplification due to its unique structure design and excellent cleaving ability. (iii) Higher reliability for quantitative detection of nucleic acids. Our real-time CHB-LAMP has high sensitivity with better quantitative ability than the conventional MB-LAMP. (iv) Easy coupling with smartphone-based point of care detection. The enhanced fluorescence caused by the CHB probe can readily be read by smartphone, enabling smartphone-based point of care diagnostics. (v) Applicability to different nucleic acid detection methods. Apart from the LAMP assay, the CHB probe has been used for other isothermal amplification, such as IMSA assay. Regarding these advantages, we have shown that CHB-enhanced fluorescence detection is beneficial for the development of simple, affordable smartphone-based point-of-care pathogen diagnostics in resource-limited settings.

## Materials and methods

### Materials and reagents

DNA staining dye 20 × EvaGreen was purchased from Biotium (Fremont, CA). Deoxynucleotide (dNTP) solution mix (10 mM of each), *Bst* 2.0 WarmStart DNA polymerase (8 U/μL), Mg_2_SO_4_ (100 mM), 10 × Isothermal Amplification Buffer (200 mM Tris–HCl, 500 mM KCl, 100 mM (NH_4_)_2_SO_4_, and 20 mM MgSO_4_, 1.0% Tween 20 and pH 8.8 at 25 °C) were purchased from New England BioLabs (Ipswich, MA). The ribonuclease RNase H2 (50 U at 2 U/μL) (Dilution Buffer included), primers, CHB and molecular beacon probes, and the pUCIDT (Amp) plasmid containing 300-bp *B. burgdorferi recA* gene sequence, or 300-bp Enterovirus 71 (EV71) VP1 gene sequence were purchased from or synthesized by Integrated DNA Technologies (Coralville, IA). DNA was extracted from each tick individually, using a MACHEREY–NAGEL nucleospin tissue kit (MACHEREY–NAGEL GmbH & Co. KG, PA, USA). Maestrogen UltraSlim LED blue light illuminator was purchased from Fisher Scientific (Pittsburgh, PA).

### CHB-LAMP assay

CHB-enhanced LAMP (CHB-LAMP) primers (F3, B3, FIP, BIP, LF and LB) for the *B. burgdorferi recA* gene sequence were designed using the online PrimerExplorer tool/software (https://primerexplorer.jp/e/). The CHB probe was designed using the online OligoAnalyzer Tool (https://www.idtdna.com/calc/analyzer) and the NUPACK platform (https://www.nupack.org/). Details of the primers and probes are shown in Supplementary Table S1. The optimal CHB-LAMP assay consisted of 1 × Isothermal Amplification Buffer, 4 mM MgSO_4_, 1.6 mM each of dNTPs, 2 U/mL RNase H2, 0.2 μM F3, 0.2 μM B3, 1.6 μM FIP, 1.6 μM BIP, 0.8 μM LF, 0.8 μM CHB, 0.32 U/μL *Bst* 2.0 WarmStart DNA polymerase, and 1 μL of the plasmid solutions or DNA samples extracted from tick species. For real-time fluorescence detection, the prepared CHB-LAMP solutions were incubated and monitored at 60 °C for 60 min in the Bio-Rad CFX96 Real-Time System. For endpoint detection, the reaction solutions in the tubes were imaged using the Bio-Rad Gel Imaging System at room temperature after 60-min incubation. The images were analyzed by ImageJ software or a customized smartphone app entitled “Fluorescence Reader”. Statistical analysis was conducted using the unpaired t-test in Prism 8 software.

### CHB-IMSA assay

CHB-enhanced IMSA (CHB-IMSA) primers (DsF, DsR, FIT, RIT, SteF and SteR) for EV71 VP1 gene sequence were obtained from previous publication^7^. The CHB probe was designed using the online OligoAnalyzer Tool (https://www.idtdna.com/calc/analyzer) and the NUPACK platform (https://www.nupack.org/). Details of the primers and probes are shown in Supplementary Table S1. The optimal CHB-IMSA assay consisted of 1 × Isothermal Amplification Buffer, 6 mM MgSO_4_, 1.4 mM each of dNTPs, 0.8 M betaine (Sigma-Aldrich), 2 U/mL RNase H2, 0.2 μM DsF, 0.2 μM DsR, 0.8 μM FIT, 0.8 μM RIT, 1.6 μM SteF, 1.2 μM SteR, 0.4 μM CHB, 0.32 U/μL *Bst* 2.0 WarmStart DNA polymerase, and 1 μL of the plasmid solutions. For real-time fluorescence detection, the prepared CHB-IMSA solution was incubated and monitored at 63 °C for 60 min in the Bio-Rad CFX96 Real-Time System.

### MB-LAMP and PCR assays

Molecular beacon-based LAMP (MB-LAMP) assay and a real-time PCR assay were run as references. The molecular beacon (Supplementary Table S1) specific to *B. burgdorferi recA* gene sequence was used as described previously^17^. The other primers used for the MB-LAMP were the same as those in the CHB-LAMP assay above. The MB-LAMP reaction system consisted of 1 × Isothermal Amplification Buffer, 4 mM MgSO_4_, 1.6 mM each of dNTPs, 0.2 μM F3, 0.2 μM B3, 1.6 μM FIP, 1.6 μM BIP, 0.8 μM MB, 0.8 μM LF, 0.32 U/μL *Bst* 2.0 WarmStart DNA polymerase, and 1 μL of the plasmid template solutions or the DNA samples extracted from tick species.

For the PCR assay, the SsoAdvanced Universal SYBR Green Supermix kit from the Bio-Rad Laboratories was used and the primers were shown in Supplementary Table S1. Based on the manufacturer’s instructions, the reaction contained 1 × Supermix, 400 nM each of primers, and 1 μL of DNA sample. The thermal cycling protocol was 2.5 min at 98 °C for initial denaturation, 35 cycles of 15 s at 95 °C for denaturation and 30 s at 60 °C for annealing and extension, followed by a melt-curve analysis (from 65 to 95 °C with 0.5 °C increment).

## Supplementary information

Supplementary Information.
