# Supplementary material for: Cleavable hairpin beacon-enhanced fluorescence detection of nucleic acid isothermal amplification and smartphone-based readout
Source: Sci Rep. 2020 Nov 2;10:18819. doi: 10.1038/s41598-020-75795-y (PMC7608614; doi:10.1038/s41598-020-75795-y)
Supplement: Supplementary file 1 — Supplementary Information. [file 41598_2020_75795_MOESM1_ESM.pdf]

Supplementary information

Cleavable Hairpin Beacon-Enhanced Fluorescence Detection of Nucleic Acid Isothermal Amplification and Smartphone-based Readout

Xiong Ding [a], Kun Yin [a], Ziyue Li [a], Vikram Pandian [b], Joan A. Smyth [c], Zeinab Helal [c] and Changchun Liu [a]*

[a] *Department of Biomedical Engineering, University of Connecticut Health Center, 263 Farmington Ave., Farmington, CT, 06030, United States*
[b] *Electrical and Computer Engineering, University of Texas at Austin, Austin, TX 78712, United States*
[c] *Connecticut Veterinary Medical Diagnostic Laboratory, Department of Pathobiology and Veterinary Science, University of Connecticut, Storrs, CT, 06269, United States*

* **Corresponding author**
Dr. Changchun Liu
Department of Biomedical Engineering
University of Connecticut Health Center
263 Farmington Avenue
Farmington, CT 06030
Phone: (860)-679-2565
E-mail: chaliu@uchc.edu

Table of Contents

Supporting Table

Table S1. Sequence information of the primers and targets used in this study.

Supporting Figures (Figure S1-S8)

Supporting Table

Table S1. Sequence information of the primers and targets used in this study.

Name	Sequence (5′-3′) [a]	Description
recA plasmid	AAGAGCATGCTCAGCATCAATAAAAGCAGCTATCCCACCT TCTTTTTGCACCTCAGCAATCGCTTGAAGAGTTAAAGTAG TCTTGCCAGACGACTCGGGGCCAAAAATTTCTATTATGCG CCCCCTAGGATATCCGCCAATGCCGAGAGCCTCATCTAAT ACAATAGATCCACTTGACATACTTTTTATACCTTGTCCAAC AGGAGATTCCCCCATCTTAATAAGACTTCCCTTTCCAAAA GCTTTTTCTATTTGAACTCTTGCAAGCTCAATAGCTTCCTC TTTACTTGCCCTTTCTAT	The 300-bp highly conversed *B. burgdorferi recA* sequence inserted into the pUCIDT (Amp) plasmid
F3_*recA*_P1	GCAGCTATCCCACCTTCT	LAMP forward outer primer targeting the *recA* gene (Primer set 1)
B3_*recA*_P1	GGAATCTCCTGTTGGACAAG	LAMP backward outer primer targeting the *recA* gene (Primer set 1)
FIP_*recA*_P1	GAAATTTTTGGCCCCGAGTCGTTTGCACCTCAGCAATCG	LAMP forward inner primer targeting the *recA* gene (Primer set 1)
BIP_*recA*_P1	TTATGCGCCCCCTAGGATATCGTATAAAAAGTATGTCAAG TGGATC	LAMP backward inner primer targeting the *recA* gene (Primer set 1)
LF_*recA*_P1	CTGGCAAGACTACTTTAACTCTTCA	LAMP forward loop primer targeting the *recA* gene (Primer set 1)
LB_*recA*_P1	CAATGCCGAGAGCCTCATCT	LAMP backward loop primer targeting the *recA* gene (Primer set 1)
F3_*recA*_P2	AGCATCAATAAAAGCAGCTATC	LAMP forward outer primer targeting the *recA* gene (Primer set 2)
B3_*recA*_P2	ATCTCCTGTTGGACAAGG	LAMP backward outer primer targeting the *recA* gene (Primer set 2)
FIP_*recA*_P2	AGTCGTCTGGCAAGACTACTTCCACCTTCTTTTTGCACCT	LAMP forward inner primer targeting the *recA* gene (Primer set 2)
BIP_*recA*_P2	AATTTCTATTATGCGCCCCCTAGAAAAAGTATGTCAAGTG GATCT	LAMP backward inner primer targeting the *recA* gene (Primer set 2)
LF_*recA*_P2	TAACTCTTCAAGCGATTGCTG	LAMP forward loop primer targeting the *recA* gene (Primer set 2)
LB_*recA*_P2	GATATCCGCCAATGCCGAGA	LAMP backward loop primer targeting the *recA* gene (Primer set 2)
CBP probe with one ribonucleotide	/56-FAM/CAATGCCGAGAGCCTCATCTAATAGCATTG/3Dabcyl/	CBP specific to the *recA* gene (for CBP-LAMP)
MB (molecular beacon)	/56-FAM/CAATGCCGAGAGCCTCATCTAATACGGCATTG/3Dabcyl/	MB specific to the *recA* gene
F-PCR_*recA*	GCAGCTATCCCACCTTCT	PCR forward primer targeting the *recA* gene
R-PCR_*recA*	GGAATCTCCTGTTGGACAAG	PCR reverse primer targeting the *recA* gene

VP1 plasmid	AACCCCTCGGTTTTTGTCAAGCTGTCAGACCCTCCATCGCAGGTTTCAGTGCCATTCATGTCACCTGCGAGTGCTTATCAATGGTTTTATGACGGATATCCCACATTCGGAGAACACAAACAGGAGAAAGATCTTGAATATGGGGCATGTCCTAATAACATGATGGGCACGTTCTCAGTGCGGACTGTGGGGACCTCCAAGTCCAAGTACCCTTTAGTGGTTAGGATTTACATGAGAATGAAGCACGTCAGGGCGTGGATACCTCGCCCGATGCGTAACCAGAACTACCTATTCAAAGCC	The 300-bp highly conversed Enterovirus 71 (EV71) VP1 inserted into the pUCIDT (Amp) plasmid
DsF_VP1	ACCATTGATAAGCACTCGCAGGGTCAAGCTGTCAGACCCTCC	IMSA forward displacement primer targeting VP1 gene
DsR_VP1	GAACACAAACAGGAGAAAGATCTTGTGAGAACGTGCCCATCA	IMSA reverse displacement primer targeting VP1 gene
FIT_VP1	TCCGAATGTGGGATATCCGTCATAAGTTTCAGTGCCATTCATGTC	IMSA forward inner primer targeting VP1 gene
RIT_VP1	TTATGACGGATATCCCACATTCGGAAGGACATGCCCCGTATT	IMSA reverse inner primer targeting VP1 gene
SteF_VP1	GAACACAAACAGGAGAAAGATCTTG	IMSA forward stem primer targeting VP1 gene
SteR_VP1	ACCATTGATAAGCACTCGCAGG	IMSA reverse stem primer targeting VP1 gene
CHB_VP1	/56-FAM/TCGCACCATTGATAAGCACTCGCAGGTGGTGCGA/3Dabcyl/	CHB specific to VP1 gene (for CHB-IMSA)

[a] Underlined bases represents the ribonucleotides

Supporting Figures

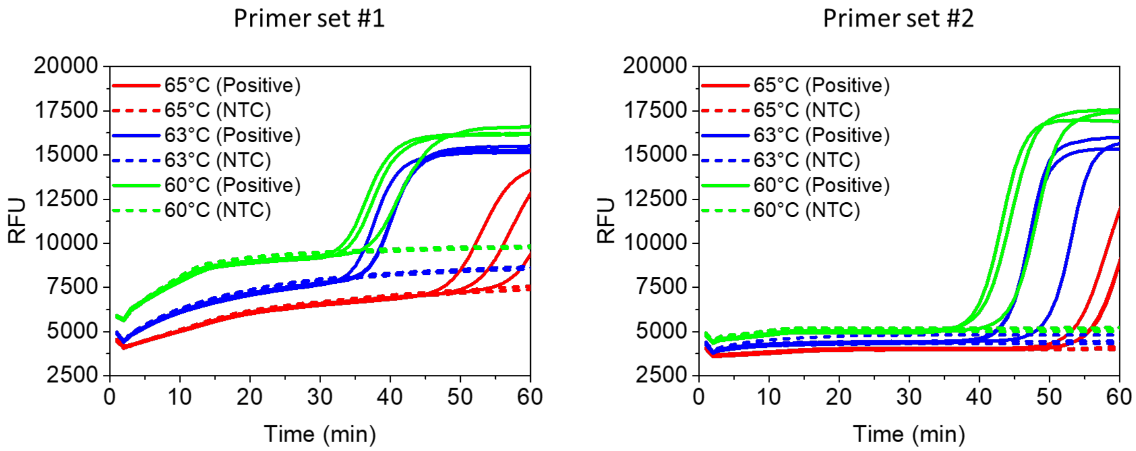

Figure S1. Comparison of two primer sets for EvaGreen-based LAMP assay for the detection of *Borrelia burgdorferi* (*B. burgdorferi*) *recA* gene sequence at various reaction temperatures. Three replicates were performed for both the positive and NTC samples. Positive, 10^5 copies of plasmid templates containing *recA* gene sequence. NTC, non-template control.

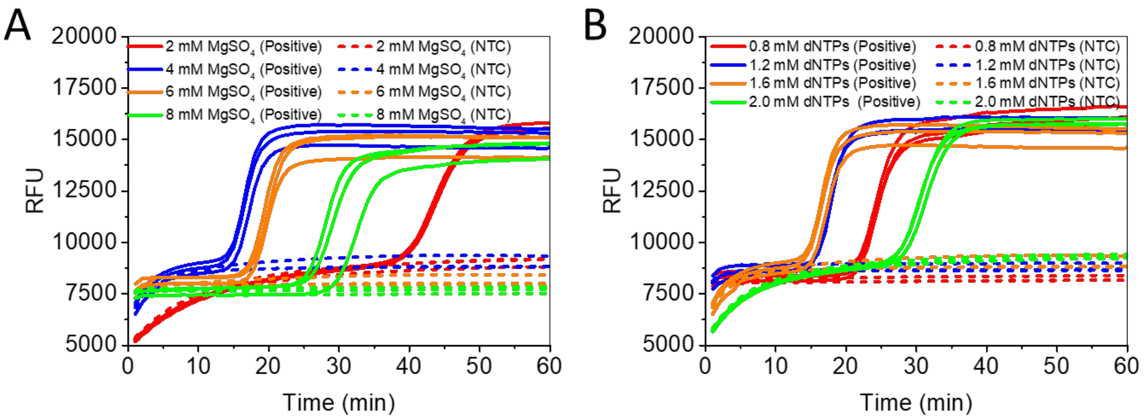

Figure S2. Optimization of the concentrations of (A) $MgSO_4$ and (B) dNTPs on EvaGreen-based LAMP assay. Primer set #1 in Figure S1 was used. Three replicates were performed for both positive and NTC samples. Positive, 10^5 copies of plasmid templates containing *recA* gene sequence. NTC, non-template control.

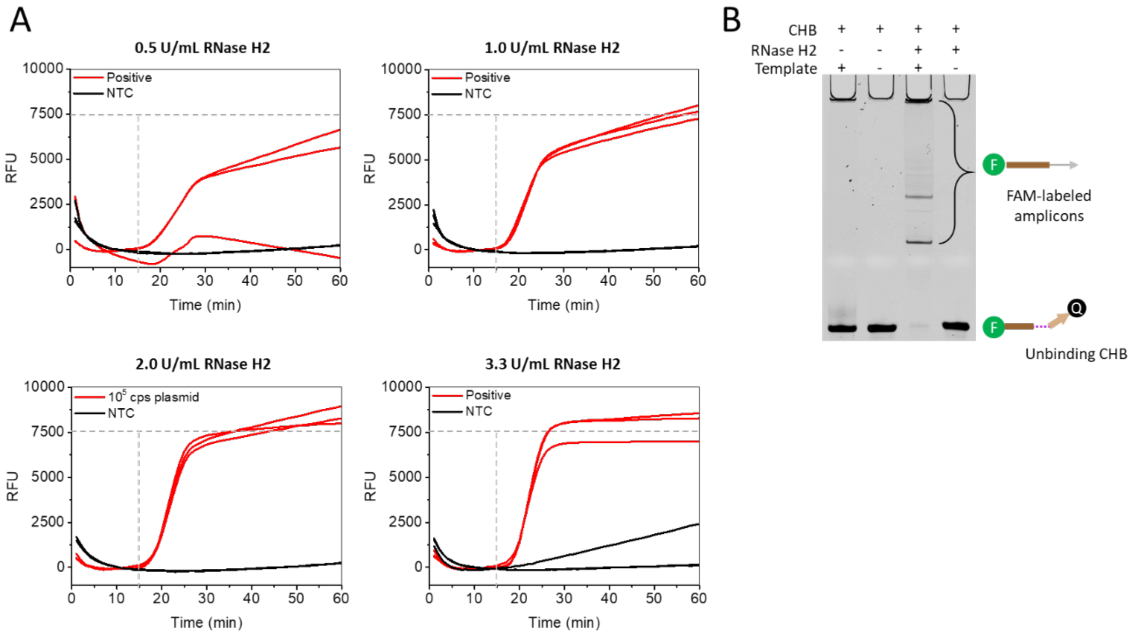

Figure S3. Optimization of the CHB-LAMP assay. (A) Real-time fluorescence curves of the CHB-LAMP assays with various RNase H2 concentrations (from 0.5 to 3.3 U/mL). Three replicates were performed for both positive and NTC samples. (B) CHB-LAMP products after denaturing PAGE. Positive, the 10-μL CHB-LAMP reaction with 10^5 copies (cps) of plasmids (300-bp *recA* gene inserted). NTC, non-template control.

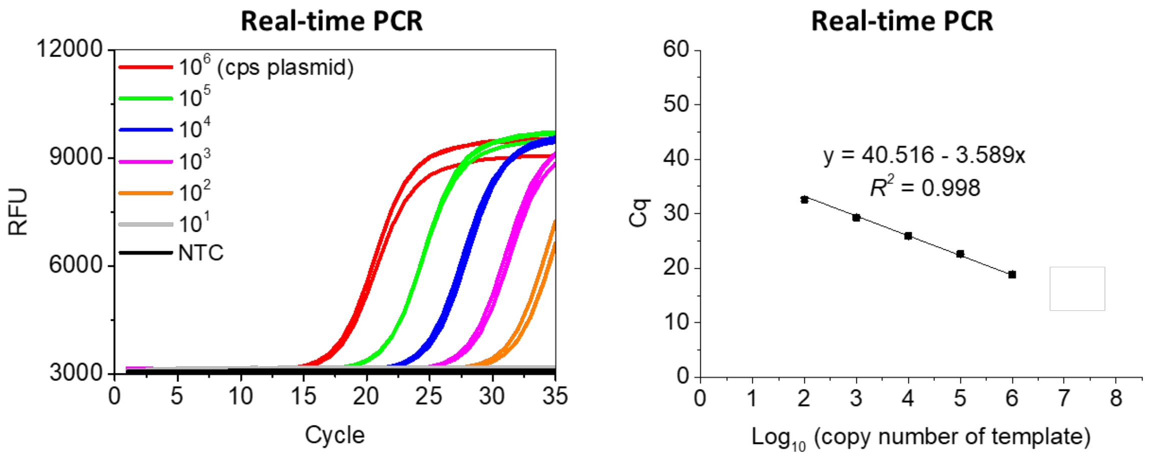

Figure S4. The sensitivity of real-time PCR assay for *B. burgdorferi* DNA detection. Left, the real-time fluorescence PCR curves; right, the linear relationship between the threshold time (Cq in PCR) and the $\log_{10}$ of template's copy number. The plasmids (*recA* gene sequence included) with the copy number (cps) ranging from 10^6 to 10^1 were used as the templates. NTC, non-template control. Each error bar represents the standard deviation for three replicates.

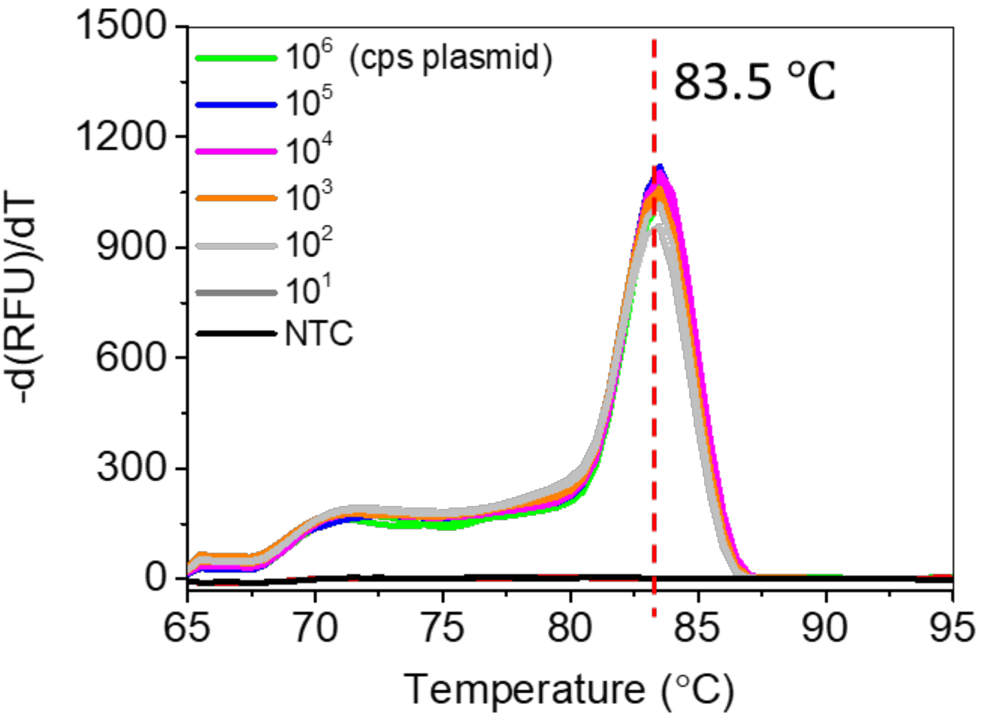

Figure S5. The melting curves for the PCR products. The melting temperature of the products was about 83.5 °C. Plasmids containing the *recA* gene sequence with copy number ranging from 10^6 to 10^1 were used as the templates. NTC, non-template control.

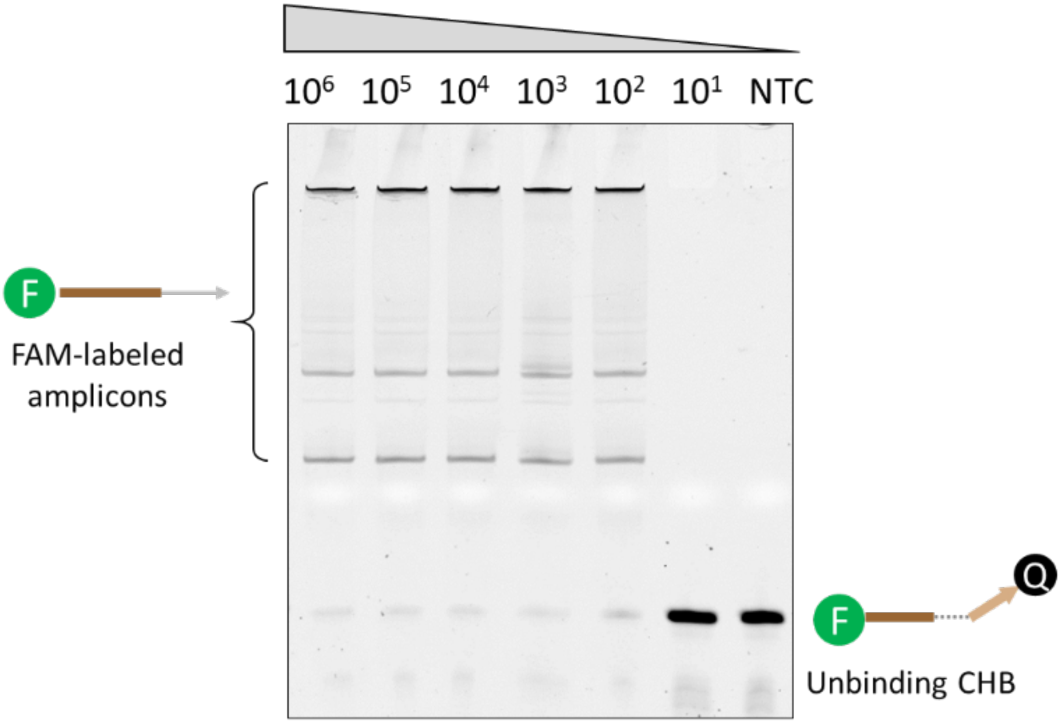

Figure S6. Product analysis of the CHB-LAMP products with RNase H2 using denaturing PAGE. The plasmids (*recA* gene sequence included) with the copy number ranging from 10^6 to 10^1 were used as the templates. NTC, non-template control.

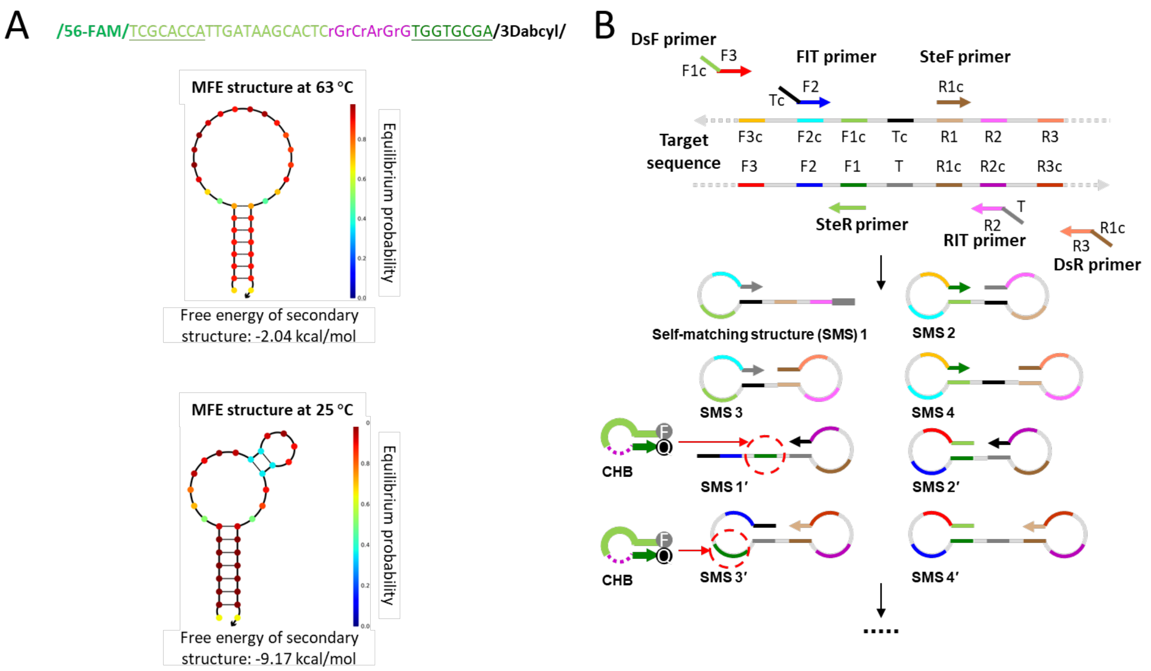

Figure S7. Principle of the CHB-IMSA assay. (A) The CHB probe sequence for the IMSA assay to amplify EV71 VP1 gene and its minimum free energy (MFE) analysis using the software NUPACK (Caltech) with concentrations of 0.4 μM CHB, 70 mM Na^{+}, and 8 mM Mg^{2+}. (B) Schematic of the CHB-IMSA assay.

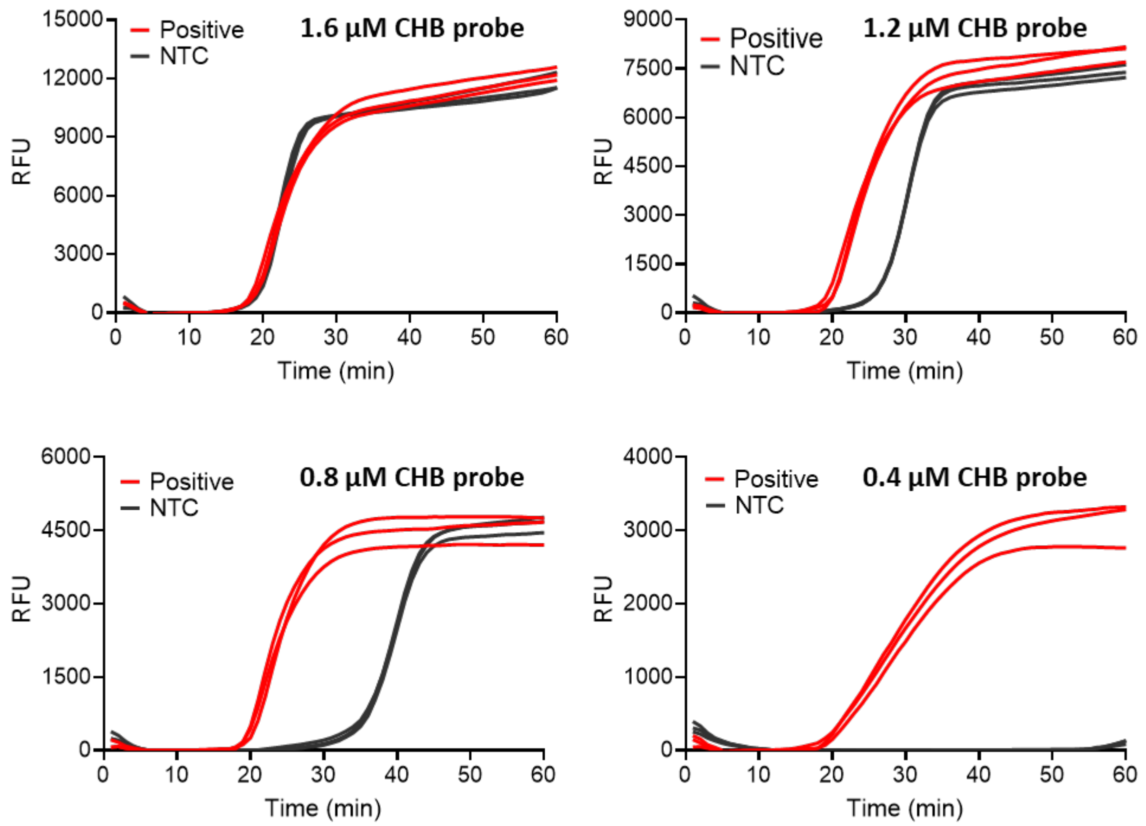

Figure S8. Optimization of the CHB probe concentration in the CHB-IMSA assay. Three replicates were set for positive and NTC. Positive, the CHB-IMSA reaction with 10^5 copies (cps) of plasmids (inserted VP1 gene). NTC, non-template control.
